# Correlation of cognitive impairment with Mediterranean diet and mortality: a prospective cohort study

**DOI:** 10.3389/fnagi.2025.1556608

**Published:** 2025-04-09

**Authors:** Ling Li, Xiaoxiao Zheng, Hongyue Ma, Mingxia Zhu, Xiuli Li, Xinhong Feng

**Affiliations:** Department of Neurology, Beijing Tsinghua Changgung Hospital, School of Clinical Medicine, Tsinghua University, Beijing, China

**Keywords:** Mediterranean diet, cognitive impairment, all-cause mortality, NHANES, cardiovascular mortality

## Abstract

**Background and aim:**

Long-term adherence to the Mediterranean Diet has been shown to improve cognitive function in patients. However, there is a lack of evidence regarding the impact of the Mediterranean diet and cognitive impairment on long-term mortality outcomes. This study aims to explore whether there is an interaction between the degree of adherence to the Mediterranean diet and cognitive impairment on long-term mortality outcomes.

**Methods:**

The study included 2,520 participants from the National Health and Nutrition Examination Survey (NHANES) conducted between 2011 and 2014. The adherence to the Mediterranean diet was assessed using the 9-point alternative Mediterranean diet index (aMED index). Cognitive function was assessed using the Consortium to Establish a Registry for Alzheimer’s disease (CERAD), the Animal Fluency Test (AFT), and the Digital Symbol Substitution Test (DSST). By accessing public records from the National Death Index (NDI), NHANES participants’ information was linked to death certificate records to determine mortality and causes of death during the follow-up period, up to December 31, 2019, with causes specified according to ICD-10. Participants were categorized based on the median aMED score into low adherence (scores 0–3), moderate adherence (score 4), and high adherence (scores 5–9) groups. Cognitive impairment was assessed by calculating the arithmetic mean of standardized scores (Z-scores) for each cognitive test. Participants with scores below the first quartile of the arithmetic mean were considered to have cognitive impairment. Cox proportional hazards regression models were used to assess the relationship between cognitive impairment, aMED, and all-cause and cardiovascular mortality outcomes. Additionally, the interaction between cognitive impairment and aMED on these outcomes was evaluated.

**Results:**

The study included 2,520 participants, with 481 deaths during the follow-up period, of which 129 (26.8%) were cardiovascular-related. The median aMED score in the population was 4, and 632 individuals (25.1%) were considered to have cognitive impairment. A higher aMED score was associated with a reduced risk of long-term all-cause mortality and cardiovascular-related mortality (HR, 0.65; 95% CI, 0.52–0.81, *p* < 0.001; HR, 0.73; 95% CI, 0.47–0.91, *p* = 0.039). Cognitive impairment was associated with an increased risk of long-term all-cause mortality and cardiovascular mortality (HR, 1.78; 95% CI, 1.46–2.18, *p* < 0.001; HR, 1.80; 95% CI, 1.22–2.64, *p* = 0.003). Individuals with both lower aMED scores and cognitive impairment had higher risks of all-cause and cardiovascular mortality. Subgroup analysis indicates that only in the cognitive impairment subgroup is a higher Mediterranean diet score associated with a reduced risk of cardiovascular mortality. There is an interaction between lower aMED scores and cognitive impairment in increasing cardiovascular-related mortality (p for interaction = 0.028).

**Conclusion:**

There is an interaction between adherence to the Mediterranean diet and cognitive impairment concerning cardiovascular-related mortality, but not all-cause mortality. Among individuals with cognitive impairment, adherence to the Mediterranean diet has a more significant impact on cardiovascular-related mortality.

## 1 Introduction

The global trend of aging is rapidly intensifying. Data shows that in 2019, the global population aged 65 and older reached 703 million. This number is projected to more than double, reaching 1.5 billion by 2050 (Economic and Affairs, 2020). As age increases, cognitive function typically declines, which is particularly concerning in our current aging society (Murman, 2015). Cognitive impairment generally includes conditions such as mild cognitive impairment (MCI) and dementia, which not only affect the quality of life of the elderly but are also closely associated with an increased risk of mortality (Bae et al., 2018). The occurrence of cognitive impairment may be related to various factors, including genetics, lifestyle, and chronic diseases (Gil-Peinado et al., 2023). Cognitive impairment is also associated with the development of cardiovascular diseases. However, there is relatively limited research on the relationship between cognitive impairment and long-term outcomes, especially all-cause and cardiovascular mortality, and the findings are somewhat controversial.

The Mediterranean diet, characterized by its emphasis on fruits, vegetables, whole grains, nuts, legumes, and fish, is rich in healthy unsaturated fats, antioxidants, and fiber. It is widely recognized for its protective effects on the cardiovascular system (Widmer et al., 2015; Aznar de la Riera et al., 2025). Numerous studies have shown that adherence to the Mediterranean diet can significantly reduce the risk of developing cardiovascular diseases (Grosso et al., 2017; Estruch et al., 2018; Esmaeilinezhad et al., 2025; Molani-Gol and Rafraf, 2025). In addition, the Mediterranean diet may beneficially influence cognitive function by improving inflammatory responses and oxidative stress, thereby reducing the risk of cognitive impairment (Frisardi et al., 2010; Cardelo et al., 2022; Pontifex et al., 2024). However, systematic research on the role of the Mediterranean diet in patients with cognitive impairment and its impact on long-term mortality risk is lacking. Given the significant association of both the Mediterranean diet and cognitive impairment with cardiovascular health, we hypothesize that within the context of cognitive impairment, the Mediterranean diet may offer stronger protective effects against all-cause and cardiovascular mortality.

This study aims to explore the interaction between cognitive impairment and the Mediterranean diet on the risks of all-cause and cardiovascular mortality. We utilized data from the National Health and Nutrition Examination Survey (NHANES), which collects health and nutrition information from a representative sample of the U.S. population and conducts long-term follow-up with participants. By analyzing NHANES data, we sought to determine whether adherence to the Mediterranean diet could reduce long-term mortality risk and to further investigate whether cognitive impairment might alter the risks of all-cause and cardiovascular mortality by influencing the effects of the Mediterranean diet.

## 2 Materials and methods

### 2.1 Population

**Figure 1:**
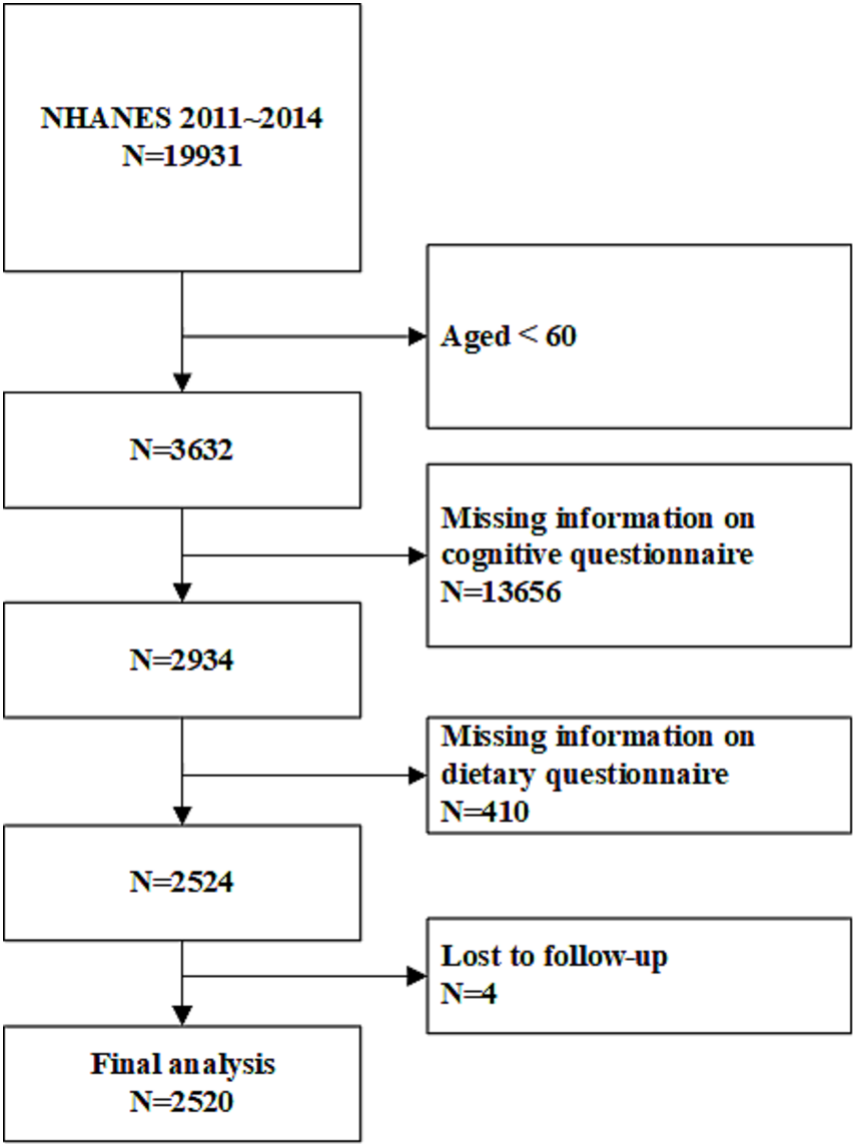
Study flowchart. NHANES, National Health and Nutrition Examination Survey. A total of 2,520 participants were included in the final analysis.

NHANES is a nationwide cross-sectional survey that assesses the health and nutritional status of the U.S. population. With a design based on complex sampling, the survey includes a population representative of all age groups and various ethnicities in the United States. Conducted in two-year cycles, NHANES collects data through interview questionnaires, standardized physical examinations, and laboratory tests (Ahluwalia et al., 2016). The survey is approved by the Institutional Review Board of the Centers for Disease Control and Prevention, with informed consent obtained from all participants.^1^ The study adheres to the principles of the Declaration of Helsinki. This analysis included data from the 2 cycles spanning 2011 to 2014. Follow-up data on mortality status and causes of death were linked to the National Death Index (up until December 31, 2019). The National Death Index, published annually by the National Center for Health Statistics (NCHS), provides epidemiologists with comprehensive mortality data for the entire U.S. population (MacMahon, 1983). The inclusion and exclusion criteria for the study population are detailed in Figure 1. Individuals younger than 60, those who did not complete the nutrition intake or cognitive questionnaires, and those lost to follow-up were excluded. Ultimately, 2,520 individuals were included in the final analysis.

### 2.2 Data collection

#### 2.2.1 Dietary assessment and aMED calculation

Relevant dietary information was obtained from the 24-h dietary recall questionnaires in the NHANES database. Participants completed the first 24-h dietary recall in a Mobile Examination Center (MEC) on Day 1, and the second 24-h dietary recall within 10 days of the MEC assessment was completed through telephone follow-up on Day 2. We used data from these two 24-h dietary recalls to estimate the average dietary intake over the 2 days.

To assess adherence to the Mediterranean diet, we employed a two-step process. First, the 24-h dietary recall data were linked to the United States Department of Agriculture (USDA) Food Patterns Equivalents Database to convert different foods and beverages into standardized food pattern components (Fan et al., 2022).

In the second step, we calculated the alternative Mediterranean diet (aMED) index to evaluate adherence to the Mediterranean diet (Fung et al., 2005). The aMED index measures the intake of 9 types of food pattern components, including total fruits, vegetables (excluding potatoes), whole grains, legumes, nuts, fish, red and processed meat, the ratio of monounsaturated to saturated fats (MUFA/SFA), and alcohol. For each type of food component, a single point will be assigned to the participants if their intake was above the median of the study cohort, except for red/processed meat and alcohol. Participants were assigned a single point for below-median intake of red/processed meat and a single point for moderate alcohol intake (defined as 10–25 g/day for men and 5–15 g/day for women). If a participant does not meet the specified condition for a given item, the score for that item is set to 0 (for example, if the participant’s fruit intake is below the median level of the total population, the score for the fruit component is 0). Finally, the scores for each item are summed to obtain the aMED score.

The total aMED score ranged from 0 to 9, with higher scores indicating greater adherence to the Mediterranean diet (Chang et al., 2022). Based on their aMED scores, participants were categorized into three groups: low adherence (aMED score 0–3), moderate adherence (aMED score 4), and high adherence (aMED score 5–9).

#### 2.2.2 Assessment of cognitive impairment

Cognitive function was assessed using three standardized tests: the Consortium to Establish a Registry for Alzheimer’s disease (CERAD) Word List Learning test, the Animal Fluency test, and the Digit Symbol Substitution Test (DSST). The CERAD Word List Learning test includes three immediate recall trials and one delayed recall trial, which evaluate the ability to learn and retain new verbal information. Each trial is scored from 0 to 10, with total possible scores ranging from 0 to 30 for the immediate recall and 0 to 10 for the delayed recall (Fillenbaum and Mohs, 2023). The Animal Fluency test measures verbal fluency within a specific category, where participants are asked to name as many animals as possible within 1 min. Scores for this test range from 0 to 40 (Lv et al., 2024). The DSST assesses cognitive domains such as processing speed, attention, and working memory by requiring participants to match symbols to numbers within a set time limit. Scores range from 0 to 133 (Tang et al., 2024). Higher scores across these tests indicate better cognitive performance. The individual scores from each test were then standardized using Z-score transformation. An overall cognitive score was calculated as the average of these standardized scores. Participants with overall cognitive scores in the lowest quartile of the study population were classified as having cognitive impairment, reflecting potential cognitive impairment.

#### 2.2.3 Assessment of other covariates

Collected baseline and sociodemographic data included age, gender, race, education level, marital status, family income poverty ratio (PIR), smoking status, BMI, and average systolic/diastolic blood pressure. Smoking was defined as smoking at least 100 cigarettes in life or smoking at the time of the survey. Laboratory tests included fasting glucose, glycated hemoglobin, total cholesterol, LDL cholesterol, HDL cholesterol, triglycerides, HOMA-IR, albumin, and eGFR. Medical condition data included histories of hypertension, diabetes, heart failure, coronary heart disease, and stroke. Hypertension history was defined as a self-reported physician diagnosis of hypertension, use of oral antihypertensive drugs, or elevated blood pressure (systolic ≥130 and/or diastolic ≥85 mmHg). Diabetes history was defined as meeting any of the following: self-reported physician diagnosis of diabetes, self-reported use of insulin or oral hypoglycemic drugs, fasting glucose concentration > 126 mg/dL, glycated hemoglobin ≥6.5%, or Oral Glucose Tolerance Test (OGTT) ≥ 200 mg/dL. Other medical histories were collected through questionnaires.

#### 2.2.4 Assessment of death

Patients were followed up until December 31, 2019. Mortality outcomes, causes of death, and follow-up time were determined based on the National Death Index as of December 31, 2019, available for download from the National Center for Health Statistics website. Causes of death were coded according to ICD-10. This study included mortality from all causes and cardiovascular disease (CVD), specifically codes I00-I09, I11, I13, and I20-I51.

### 2.3 Statistical analysis

All analyses were conducted using the R statistical package (R Foundation).^2^ This study followed the NHANES data usage guidelines for statistical analysis, taking into account the complex survey design factors.^3^ Using conventional estimations is inappropriate; thus, all analyses were weighted to represent the U.S. population. We calculated weighted estimates according to the NHANES analytic guidelines.^4^ Participants were divided into above-median, median, and below-median groups based on aMED scores, and baseline data for these groups were described. Continuous variables were presented as means (95% confidence intervals). Categorical variables were expressed as numbers (percentages). Differences in continuous variables among groups were analyzed using ANOVA with *p*-values obtained by survey-weighted linear regression (svyglm); differences in categorical variables were analyzed with the Chi-square test, with p-values from the survey-weighted Chi-square test (svytable). Cox proportional hazards regression models were used to calculate hazard ratios (HR) and 95% confidence intervals for cognitive impairment, aMED on all-cause and cardiovascular mortality. Further analysis grouped according to aMED and cognitive impairment (presence or absence) was conducted using Cox proportional hazards models to explore the relationship with mortality outcomes. The interaction effect of cognitive impairment with aMED on outcomes was assessed by the likelihood ratio test, considering two-sided interaction *p*-values of 0.1 as statistically significant. Kaplan–Meier survival curves were plotted for groups based on aMED and cognitive impairment, and compared using the log-rank test. Additionally, restricted cubic spline plots were used to evaluate the non-linear relationship between aMED scores and mortality outcomes. All analyses were repeated with complete data to examine the robustness of the results. Two-sided p-values of 0.05 were considered statistically significant.

## 3 Results

### 3.1 Baseline characteristics

A total of 2,520 participants were included in the study, categorized into low (0–3), median (4), and high (5–9) aMED score groups. The mean age of participants was 69.1 years (95% CI, 68.6–69.6), with no significant difference between groups (*p* = 0.6499). Female participants comprised 53.2% of the cohort, with a slightly higher percentage in the high aMED group (55.3%) compared to the low aMED group (50.6%), though this difference was not statistically significant (*p* = 0.3545).

There were significant differences in race distribution (*p* = 0.0019), with a higher proportion of non-Hispanic White participants in the high aMED group (77.4%) compared to the low aMED group (69.6%). Participants in the high aMED group also had a significantly higher family income-to-poverty ratio (*p* < 0.0001) and higher levels of education, with more participants having a college degree or above (41.3%) compared to the low aMED group (25.8%) (*p* < 0.0001).

Regarding lifestyle factors, participants in the high aMED group had lower smoking rates (13.3%) compared to the low aMED group (19.2%) (*p* = 0.0024). BMI was also significantly lower in the high aMED group (mean 28.3) than in the low aMED group (mean 29.7) (*p* = 0.0046). Additionally, participants in the high aMED group had lower fasting serum glucose levels (*p* = 0.0049), lower systolic blood pressure (*p* = 0.0464), and reported higher levels of physical activity (*p* = 0.0244) compared to the low aMED group (Table 1).

**Table 1:** Baseline demographics among patients with low, median, and high aMED.

Characteristics	Total	aMED (above median)	aMED (Median)	aMED (below median)	P*	P**
		Score 0–3	Score 4	Score 5–9		
Number of subjects	2, 520	955	571	994		
Age (year)	69.1 (68.6, 69.6)	69.3 (68.7, 69.9)	68.8 (67.8, 69.8)	69.1 (68.3, 69.9)	0.6499	0.6311
Female	53.2 (51.0, 55.5)	50.6 (46.2, 55.0)	53.7 (48.2, 59.1)	55.3 (50.7, 59.9)	0.3545	0.1581
Race					0.0019	0.0013
Mexican American	3.4 (2.1, 5.5)	3.0 (1.4, 6.0)	3.3 (2.0, 5.4)	3.8 (2.4, 6.1)		
Other Hispanic	4.0 (2.6, 5.9)	3.9 (2.2, 6.8)	4.3 (2.5, 7.1)	3.8 (2.6, 5.4)		
Non-Hispanic White	79.1 (74.7, 82.9)	81.0 (74.8, 85.9)	78.9 (72.7, 84.1)	77.4 (72.5, 81.7)		
Non-Hispanic Black	8.5 (6.2, 11.5)	9.3 (6.4, 13.3)	9.3 (6.3, 13.5)	7.3 (5.2, 10.3)		
Other Race	5.1 (4.1, 6.3)	2.9 (1.9, 4.2)	4.2 (2.5, 7.0)	7.6 (5.6, 10.2)		
Family PIR	3.1 (3.0, 3.3)	2.8 (2.6, 3.0)	3.1 (2.7, 3.4)	3.5 (3.3, 3.7)	<0.0001	<0.0001
Educational level					<0.0001	<0.0001
Less than 9th grade	5.5 (4.1, 7.4)	7.4 (4.9, 11.2)	5.7 (3.9, 8.3)	3.6 (2.6, 4.9)		
9–11th grade	9.5 (7.1, 12.5)	11.6 (8.7, 15.3)	10.6 (7.5, 14.9)	7.0 (4.5, 10.7)		
High school graduate	22.3 (18.7, 26.4)	26.4 (21.2, 32.3)	25.1 (18.0, 33.9)	17.0 (13.4, 21.2)		
College	30.1 (26.9, 33.5)	31.4 (25.5, 38.0)	26.0 (20.9, 31.9)	31.2 (26.3, 36.6)		
College graduate or above	32.6 (28.0, 37.7)	23.2 (18.1, 29.3)	32.5 (24.5, 41.6)	41.3 (35.1, 47.7)		
Marital status					0.0001	0.0004
Married	66.7 (63.4, 69.9)	60.7 (54.5, 66.6)	67.2 (61.4, 72.6)	71.8 (68.2, 75.2)		
Never married	3.7 (2.9, 4.8)	4.4 (3.1, 6.2)	1.9 (1.0, 3.4)	4.2 (2.8, 6.1)		
Widowed/Divorced/Separated	29.6 (26.6, 32.8)	34.9 (29.4, 40.9)	30.9 (25.7, 36.6)	24.0 (21.0, 27.4)		
Smoking	19.0 (16.9, 21.4)	26.6 (21.9, 31.8)	16.5 (11.2, 23.5)	13.3 (9.1, 19.0)	0.0024	0.0015
BMI	29.1 (28.6, 29.6)	30.1 (29.4, 30.8)	29.1 (28.0, 30.3)	28.3 (27.7, 28.8)	0.0046	0.0011
CHD	9.9 (7.9, 12.4)	11.7 (9.5, 14.4)	8.4 (4.2, 16.1)	9.1 (6.4, 12.8)	0.4202	0.1848
Stroke	6.0 (5.0, 7.1)	7.7 (5.7, 10.3)	5.7 (3.7, 8.7)	4.6 (3.0, 6.9)	0.1283	0.076
Diabetes mellitus	25.2 (22.8, 27.7)	29.5 (24.7, 34.9)	23.8 (17.7, 31.1)	22.0 (19.1, 25.2)	0.0685	0.0133
Hypertension	73.9 (70.7, 76.8)	76.8 (69.4, 82.9)	72.0 (65.7, 77.6)	72.3 (68.5, 75.7)	0.3813	0.2269
Cognitive impairment	19.1 (17.1, 21.3)	23.8 (20.9, 26.9)	16.6 (12.9, 21.0)	16.4 (13.3, 20.0)	0.0014	0.0009
TG (mmol/L)	1.4 (1.3, 1.5)	1.5 (1.4, 1.6)	1.3 (1.2, 1.4)	1.4 (1.2, 1.5)	0.0139	0.0505
TC (mmol/L)	5.0 (4.9, 5.1)	4.9 (4.8, 5.1)	5.0 (4.8, 5.2)	5.0 (4.9, 5.1)	0.6827	0.43
HDL-C (mmol/L)	1.4 (1.4, 1.5)	1.4 (1.3, 1.4)	1.4 (1.3, 1.5)	1.5 (1.5, 1.6)	0.0027	0.0007
LDL-C (mmol/L)	2.9 (2.8, 2.9)	2.8 (2.7, 2.9)	2.9 (2.7, 3.1)	2.9 (2.8, 3.0)	0.5872	0.311
Fasting glucose (mmol/L)	6.2 (6.0, 6.4)	6.3 (6.1, 6.6)	6.5 (5.8, 7.1)	6.0 (5.8, 6.1)	0.0464	0.0188
HbA1c, %	5.9 (5.9, 6.0)	6.0 (5.9, 6.1)	5.9 (5.8, 6.0)	5.9 (5.8, 5.9)	0.1311	0.0484
HOMA-IR	4.0 (3.4, 4.6)	4.1 (3.2, 4.9)	4.9 (3.4, 6.4)	3.5 (3.0, 3.9)	0.1842	0.2383
Albumin	42.0 (41.8, 42.2)	41.7 (41.3, 42.0)	42.2 (41.8, 42.5)	42.3 (41.9, 42.6)	0.0244	0.0122
eGFR, mL/min	72.1 (70.8, 73.5)	69.5 (66.2, 72.8)	71.5 (69.1, 73.9)	74.8 (73.5, 76.1)	0.0049	0.0063
CERAD W-L immediate recall	19.8 (19.4, 20.3)	19.1 (18.5, 19.7)	19.9 (19.2, 20.6)	20.4 (19.9, 20.9)	0.0005	0.0001
CERAD W-L delayed recall	6.3 (6.1, 6.5)	6.1 (5.8, 6.4)	6.4 (6.0, 6.7)	6.6 (6.3, 6.8)	0.0568	0.0197
Animal fluency test	18.2 (17.7, 18.6)	17.0 (16.4, 17.6)	18.3 (17.6, 19.0)	19.1 (18.5, 19.7)	<0.0001	<0.0001
Digit symbol substitution test	52.6 (51.3, 53.8)	49.0 (47.5, 50.6)	52.8 (50.3, 55.3)	55.6 (53.8, 57.4)	<0.0001	<0.0001

*Comparison among three groups. **Comparison between aMED Below Median and above median. aMED, alternative Mediterranean diet; PIR, poverty income ratio; BMI, body mass index; SBP, systolic pressure; DBP, diastolic pressure; CHF, congestive heart failure; CHD, coronary heart disease; TG, triglycerides; TC, total cholesterol; HDL-C, high density lipoprotein-cholesterol; LDL-C, low density lipoprotein-cholesterol; HbA1c, glycated hemoglobin A1c; HOMA-IR, Homeostatic Model Assessment for Insulin Resistance; eGFR, estimated glomerular filtration rate. Data are presented as mean (95% CI) or percentage (95% CI). For continuous variables, *p*-value was by survey-weighted linear regression (svyglm). For categorical variables, *p*-value was by survey-weighted Chi-square test (svytable).

A total of 19.1% participants were categorized as having cognitive impairment. Participants with cognitive impairment were older, had lower PIR, higher rate of CHD, stroke and diabetes, have higher level of HbA1c, and have lower level of TC, LDL-C, and eGFR. There were no significant differences in TG and HDL-C levels between the cognitive impairment group and the non-cognitive impairment group. Males, the widowed, the divorced or the separated had higher risk of cognitive impairment (Supplementary Table 1).

### 3.2 Association between aMED, cognitive impairment, and mortality

#### 3.2.1 All-cause mortality

In the unadjusted model, higher aMED scores were associated with a significantly lower risk of all-cause mortality. Participants in the high aMED group had a hazard ratio (HR) of 0.56 (95% CI 0.45–0.69, *p* < 0.001) compared to the reference low aMED group. The median aMED group also showed a reduced risk of all-cause mortality (HR 0.73, 95% CI 0.58–0.92, *p* = 0.007). When aMED was analyzed as a continuous variable, there was a consistent protective effect (HR 0.86, 95% CI 0.81–0.91, *p* < 0.001). Confounders were chosen from univariate regression analysis and clinical experience. To detect multicollinearity of the confounders, we calculated Variance Inflation Factors (VIFs) for all covariates included in the adjusted Cox regression model. The VIF values ranged between 1.0 and 1.5 (Supplementary Table 2). These results suggest that multicollinearity among covariates is negligible in our model, and therefore does not significantly inflate standard errors or destabilize hazard ratio estimates. After adjusting for confounding variables (Model III), the protective effect of a high aMED score remained significant (HR 0.65, 95% CI 0.52–0.81, *p* < 0.001). The association for the median aMED group, however, was attenuated and no longer significant (HR 0.82, 95% CI 0.64–1.05, *p* = 0.115). Cognitive impairment was strongly associated with an increased risk of all-cause mortality in both unadjusted (HR 2.80, 95% CI 2.34–3.35, *p* < 0.001) and adjusted models (HR 1.78, 95% CI 1.46–2.18, p < 0.001).

#### 3.2.2 Cardiovascular mortality

For CVD mortality, in the unadjusted model, the high aMED group had a significantly lower risk compared to the low aMED group (HR 0.60, 95% CI 0.40–0.89, *p* = 0.011), while the median aMED group showed a non-significant reduction in risk (HR 0.76, 95% CI 0.49–1.18, *p* = 0.221). aMED as a continuous variable was associated with a lower risk of CVD mortality (HR 0.77, 95% CI 0.63–0.94, *p* = 0.002).

In the fully adjusted model (Model III), the protective effect of a high aMED score persisted (HR 0.73, 95% CI 0.47–0.91, *p* = 0.039), whereas the association for the median aMED group was not significant (HR 0.88, 95% CI 0.56–1.35, *p* = 0.597). Cognitive impairment was associated with a higher risk of CVD mortality, both in the unadjusted model (HR 2.99, 95% CI 2.12–4.23, *p* < 0.001) and in the fully adjusted model (HR 1.80, 95% CI 1.22–2.64, *p* = 0.003) (Table 2).

**Table 2:** Association between aMED, cognitive impairment and mortality.

Mortality outcomes	Unadjusted		Model I		Model II		Model III	
	HR (95% CI)	*P*-value	HR (95% CI)	*P*-value	HR (95% CI)	*P*-value	HR (95% CI)	*P*-value
All-cause mortality								
Lower aMED	Reference							
Median aMED	0.73 (0.58–0.92)	0.007	0.82 (0.65–1.03)	0.095	0.83 (0.65–1.06)	0.0001	0.82 (0.64–1.05)	0.115
Higher aMED	0.56 (0.45–0.69)	<0.001	0.57 (0.46–0.7)	<0.001	0.64 (0.51–0.8)	<0.0001	0.65 (0.52–0.81)	<0.001
aMED (continuous)	0.86 (0.81–0.91)	<0.001	0.87 (0.82–0.92)	<0.001	0.9 (0.85–0.96)	0.001	0.9 (0.84–0.96)	0.001
Cognitive impairment*	2.8 (2.34–3.35)	<0.001	1.86 (1.53–2.25)	<0.001	1.9 (1.55–2.32)	<0.001	1.78 (1.46–2.18)	<0.001
CVD mortality								
Lower aMED	Reference							
Median aMED	0.76 (0.49–1.18)	0.221	0.86 (0.55–1.35)	0.522	0.95 (0.6–1.23)	0.217	0.88 (0.56–1.35)	0.597
Higher aMED	0.6 (0.4–0.89)	0.011	0.62 (0.42–0.93)	0.022	0.75 (0.49–0.92)	0.042	0.73 (0.47–0.91)	0.039
aMED (continuous)	0.77 (0.63–0.94)	0.002	0.85 (0.77–0.95)	0.005	0.91 (0.81–1.02)	0.101	0.9 (0.8–1.01)	0.082
Cognitive impairment*	2.99 (2.12–4.23)	<0.001	1.86 (1.29–2.69)	0.001	1.92 (1.31–2.8)	0.001	1.8 (1.22–2.64)	0.003

CVD, cardiovascular disease; Model I initially was adjusted for age, sex, and race (Mexican American, non-Hispanic white, non-Hispanic black, other races); model II was further adjusted for education level (below high school, high school or equivalent, college or above), marital status (married, widowed, divorced, and never married), and family PIR based on model I; model III further adjusted for BMI, smoking status, comorbidities including diabetes mellitus, hypertension, and stroke. *No cognitive impairment was set as the reference group.

### 3.3 Interaction between aMED and cognitive impairment on mortality

In participants without cognitive impairment, the unadjusted hazard ratio (HR) for all-cause mortality was significantly higher in the low aMED group compared to the high aMED group (HR 1.63, 95% CI 1.23–2.17, *p* = 0.001). After adjusting for confounding factors, the risk remained elevated in the low aMED group, but with reduced significance (HR 1.30, 95% CI 0.96–1.77, *p* = 0.09). In participants with cognitive impairment, the risk of all-cause mortality was even higher in the low aMED group, with an unadjusted HR of 1.76 (95% CI 1.29–2.40, p < 0.001). The association remained significant after adjustment, with an adjusted HR of 1.78 (95% CI 1.25–2.52, p = 0.001). There was no significant interaction between aMED and cognitive impairment in influencing all-cause mortality (p for interaction = 0.408).

For cardiovascular (CVD) mortality, participants without cognitive impairment in the low aMED group had a higher but not statistically significant unadjusted risk (HR 1.10, 95% CI 0.62–1.93, *p* = 0.749) compared to the high aMED group. After adjustment, the HR remained non-significant (HR 0.76, 95% CI 0.41–1.40, *p* = 0.379).

In participants with cognitive impairment, the low aMED group had a significantly higher risk of CVD mortality in both the unadjusted (HR 2.22, 95% CI 1.22–4.06, *p* = 0.009) and adjusted models (HR 2.00, 95% CI 1.02–3.95, *p* = 0.045). The interaction between aMED and cognitive impairment was significant for CVD mortality (p for interaction = 0.028), indicating that cognitive impairment and low aMED scores together significantly increased the risk of cardiovascular mortality (Table 3).

**Table 3:** Interaction analysis of aMED and cognitive impairment on mortality.

aMED categories	Cognitive impairment	Unadjusted HR 95% CI	*P*-value	P for interaction	Adjusted HR 95% CI	*P*-value	*P* for interaction
*All-cause mortality*				0.768			0.408
Higher aMED	Without				1 (Ref)		
Median aMED	Without	1.36 (0.98–1.88)	0.066		1.22 (0.86–1.72)	0.261	
Lower aMED	Without	1.63 (1.23–2.17)	0.001		1.3 (0.96–1.77)	0.090	
Higher aMED	With				1 (Ref)		
Median aMED	With	1.23 (0.83–1.83)	0.294		1.41 (0.91–2.17)	0.124	
Lower aMED	With	1.76 (1.29–2.4)	<0.001		1.78 (1.25–2.52)	0.001	
*CVD mortality*				0.056			0.028
Higher aMED	Without	1 (Ref)			1 (Ref)		
Median aMED	Without	1.41 (0.78–2.54)	0.253		1.31 (0.71–2.4)	0.391	
Lower aMED	Without	1.1 (0.62–1.93)	0.749		0.76 (0.41–1.4)	0.379	
Higher aMED	With	1 (Ref)			1 (Ref)		
Median aMED	With	1.03 (0.45–2.36)	0.938		0.97 (0.38–2.46)	0.951	
Lower aMED	With	2.22 (1.22–4.06)	0.009		2 (1.02–3.95)	0.045	

Adjusted for age, sex, race (Mexican American, non-Hispanic white, non-Hispanic black, other races), education level (below high school, high school or equivalent, college or above), marital status (married, widowed, divorced, and never married), family PIR, BMI, smoking status, comorbidities including diabetes mellitus, hypertension, and stroke.

### 3.4 Sensitivity analysis

**Figure 2:**
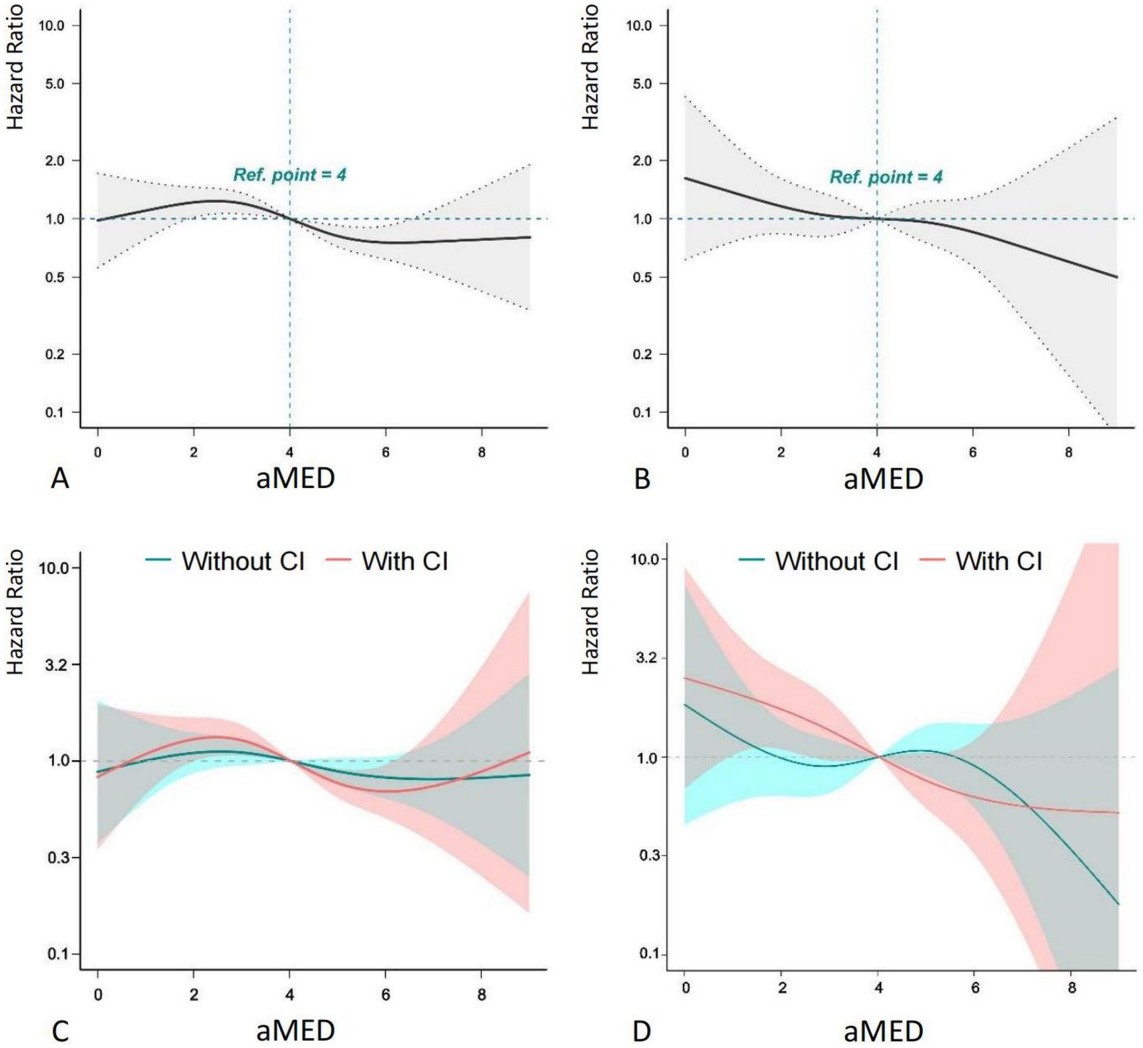
RCS plot of aMED, cognitive impairment and mortality. aMED, alternative Mediterranean Diet; HR, hazard ratio; CI, cognitive impairment. Higher aMED scores show a monotonic dose–response association with reduced all-cause mortality risk.

Further analysis using RCS curves evaluated the nonlinear relationship between aMED and both all-cause and cardiovascular mortality (Figure 2). A clear monotonic dose–response relationship was observed between aMED and all-cause mortality, indicating that higher aMED scores were associated with a lower risk of mortality. A similar linear relationship was found between aMED and cardiovascular mortality, although this association was not statistically significant. When participants were stratified by the presence or absence of cognitive impairment, the RCS curves for all-cause mortality were similar across the groups. However, for cardiovascular mortality, among those with cognitive impairment, aMED scores below 4 were associated with a more pronounced reduction in cardiovascular mortality risk compared to those without cognitive impairment. This suggests that the protective effect of a higher aMED score on cardiovascular mortality is more evident in participants with cognitive impairment.

**Figure 3:**
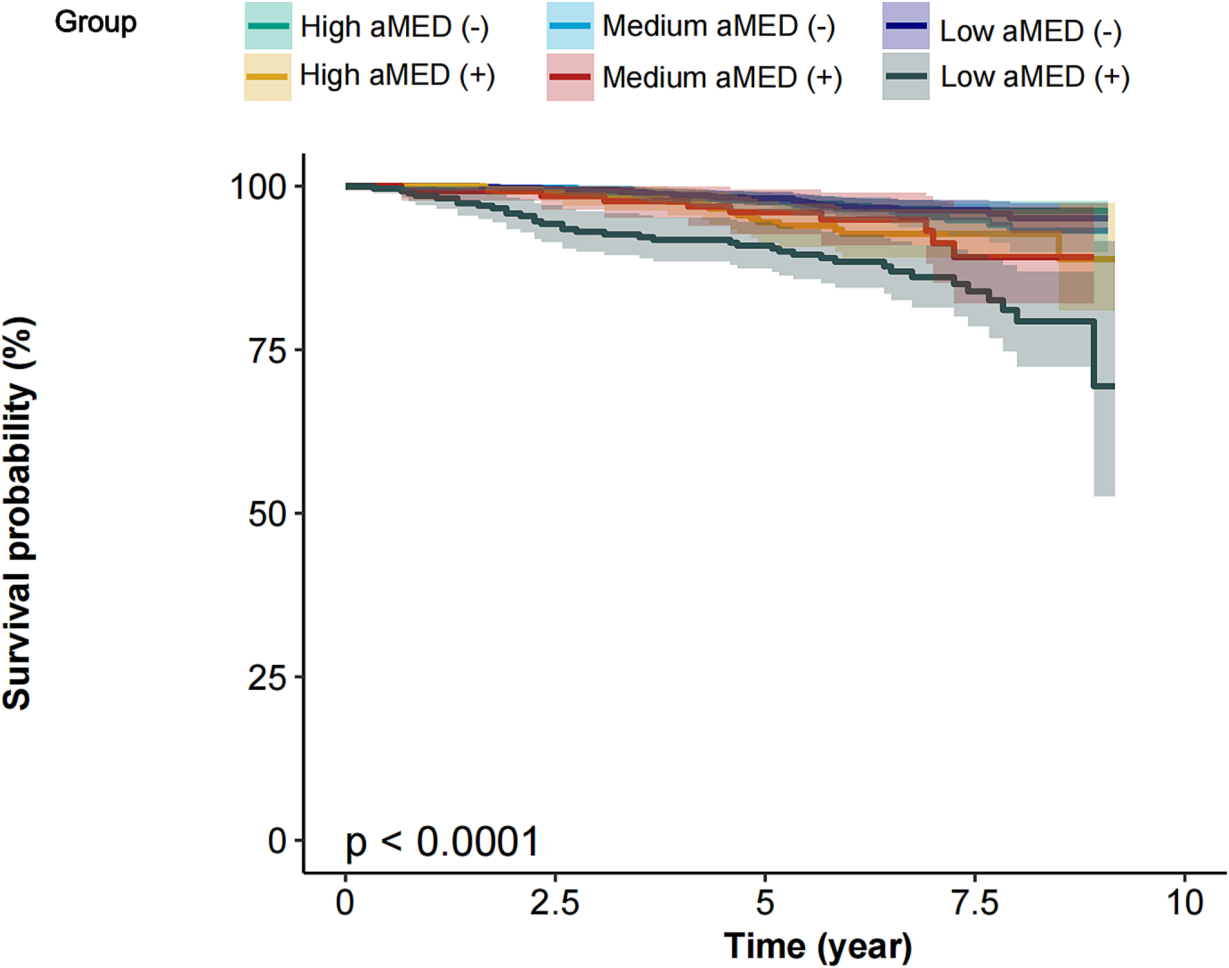
Survival curves of participants stratified by aMED scores and the presence of cognitive impairment. aMED, alternative Mediterranean Diet; High aMED (−), high aMED score without cognitive impairment; Medium aMED (−), medium aMED score without cognitive impairment; Low aMED (−), low aMED score without cognitive impairment; High aMED (+), high aMED score with cognitive impairment; Medium aMED (+), medium aMED score with cognitive impairment; Low aMED (+), low aMED score with cognitive impairment. The Low aMED (+) group had the poorest survival outcomes, suggesting that low aMED scores combined with cognitive impairment are associated with a significantly increased mortality risk.

The Kaplan–Meier survival curves stratified by aMED scores and the presence of cognitive impairment (CI) demonstrate a clear difference in survival probability across groups (*p* < 0.0001). Participants were categorized into three aMED score groups: High aMED, Medium aMED, and Low aMED. Each group was further divided into those without cognitive impairment (−) and with cognitive impairment (+), resulting in six groups: High aMED (−), Medium aMED (−), Low aMED (−), High aMED (+), Medium aMED (+), and Low aMED (+) across all groups, participants with high aMED scores exhibited the highest survival probability over the 10-year follow-up period, regardless of cognitive impairment status. In contrast, participants with low aMED scores had the lowest survival probability, particularly those with cognitive impairment [Low aMED (+)], who experienced a steep decline in survival compared to other groups. Participants with cognitive impairment generally had lower survival probabilities within each aMED category. Notably, the Low aMED (+) group had the poorest survival outcomes, suggesting that low aMED scores combined with cognitive impairment are associated with a significantly increased mortality risk (Figure 3).

**Figure 4:**
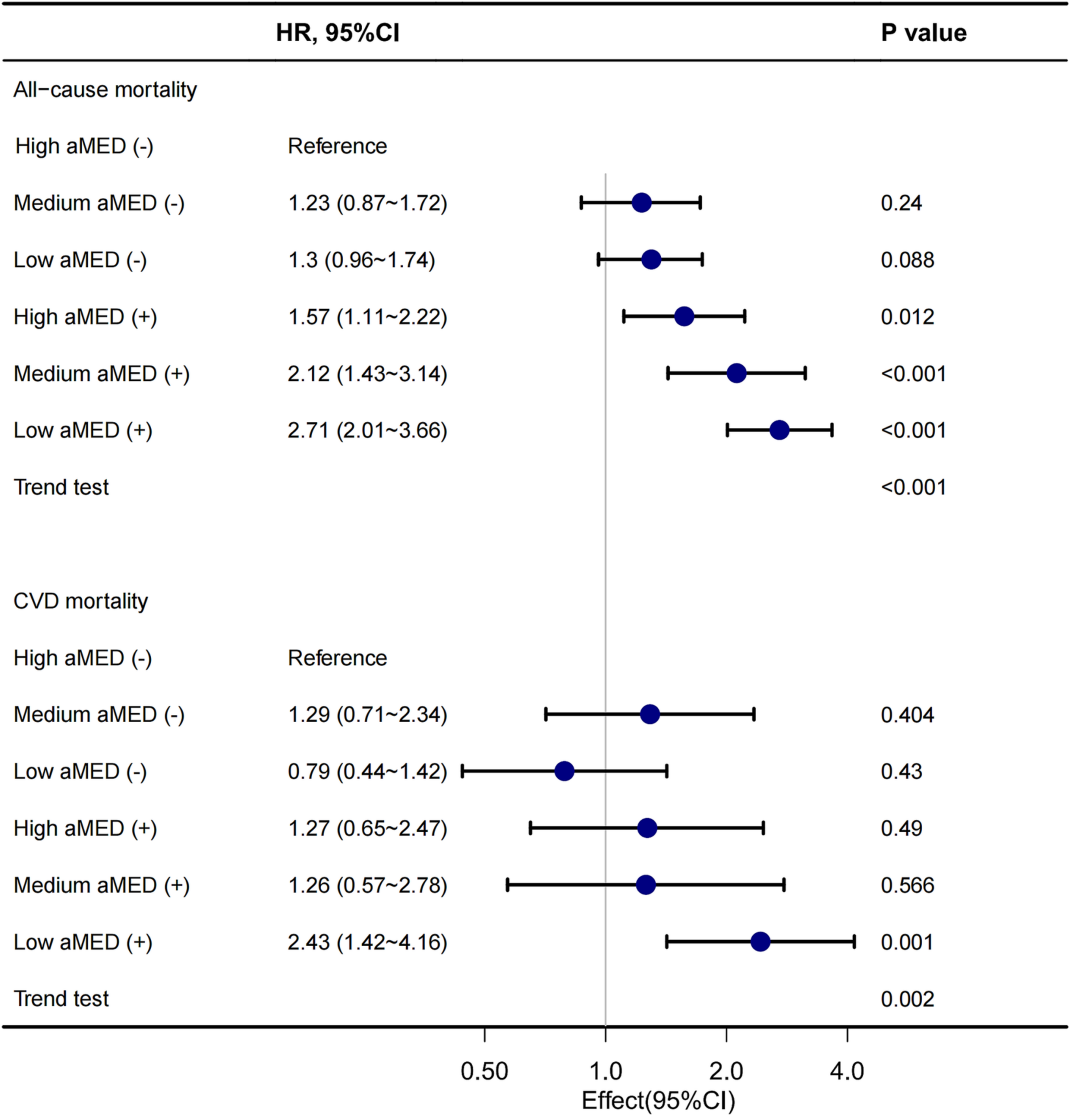
Forest plot of association between aMED, cognitive impairment and mortality. aMED, alternative Mediterranean Diet; HR, hazard ratio; CVD, cardiovascular disease. High aMED (−), high aMED score without cognitive impairment; Medium aMED (−), medium aMED score without cognitive impairment; Low aMED (−), low aMED score without cognitive impairment; High aMED (+), high aMED score with cognitive impairment; Medium aMED (+), medium aMED score with cognitive impairment; Low aMED (+), low aMED score with cognitive impairment. The stratified forest plot demonstrated that individuals with cognitive impairment and low adherence to the aMED had a 2.71-fold higher risk of all-cause mortality compared to those with high adherence, with a significant dose-dependent trend across ascending aMED categories. A consistent risk gradient was observed for CVD mortality.

A forest plot also revealed that in the presence of cognitive impairment, the risk of all-cause mortality was notably higher. The HR for Low aMED (+) compared to High aMED (−) was 2.71 (95% CI 2.01–3.66, *p* < 0.001), and a significant trend was observed across all aMED categories (p for trend <0.001). A similar trend was observed for CVD mortality (Figure 4).

## 4 Discussion

Our study identifies a critical interaction between Mediterranean diet adherence and cognitive impairment in modulating cardiovascular mortality risk (*p* for interaction = 0.028). This finding implies that the protective effect of the Mediterranean diet on cardiovascular outcomes is amplified in individuals with cognitive impairment, a population traditionally excluded from dietary intervention trials. Mechanistically, this synergy may arise from two pathways: (1) Mitochondrial and vascular benefits: the Mediterranean diet’s high antioxidant content (e.g., polyphenols, omega-3 fatty acids) may counteract oxidative stress and endothelial dysfunction—processes exacerbated in cognitive impairment due to neurodegenerative and vascular comorbidities (Caturano et al., 2025; Ilari et al., 2025). (2) Behavioral mediation: cognitive impairment often correlates with poor dietary choices and metabolic dysregulation (Młynarska et al., 2024). Adherence to the Mediterranean diet may mitigate these downstream effects, disproportionately benefiting this high-risk group. Clinically, our results advocate for prioritizing dietary interventions in cognitively impaired older adults, who face elevated mortality risk yet are rarely targeted in preventive guidelines.

The Mediterranean diet, characterized by its abundance of plant-based foods and minimally processed ingredients, has been associated with a reduced risk of various chronic diseases and increased longevity. Although the precise mechanisms by which the Mediterranean diet promotes health are not yet fully understood, a growing body of research suggests that this dietary pattern may exert its effects through multiple pathways. These include lowering blood lipids, countering oxidative stress, reducing inflammation and platelet aggregation, and regulating hormones and growth factors related to cancer development (Tosti et al., 2018). In previous cohort studies and randomized controlled trials, the Mediterranean diet has been shown to have a significant beneficial impact on all-cause mortality, cardiovascular risk factors, and the incidence and mortality of cardiovascular diseases (CVD) (Guasch-Ferré and Willett, 2021). A systematic review and dose–response meta-analysis showed that for every 2-point increase in the Mediterranean diet score, the risk of all-cause mortality decreased by 10% (95% CI: 9–11%) (Soltani et al., 2019). In patients with cardiovascular disease, higher adherence to the Mediterranean diet is associated with a reduced risk of all-cause mortality (Tang et al., 2021). Additionally, clinical trials indicate that adhering to the MD can improve endothelial function in patients with coronary heart disease (Yubero-Serrano et al., 2020) and reduces low-density lipoprotein (LDL) cholesterol related to atherosclerosis in individuals at high cardiovascular risk (Hernáez et al., 2017). Thus, it demonstrates the potential to reduce the risk of cardiovascular mortality. Our results are consistent with previous studies. Using NHANES data from the U.S. population, we found a linear association between stricter adherence to the MD and a reduced long-term risk of all-cause and cardiovascular-related mortality in the general population.

Cognitive impairment is a chronic condition that affects a person’s ability to remember, learn, focus, and make decisions in daily life (Mitchell et al., 2010). Due to changes in the global age structure, the increase in the number of individuals with dementia is a major public health concern. By 2050, the prevalence is expected to double (Livingston, 2017). The presence of cognitive impairment has been shown to be an independent predictor of mortality (Sachs et al., 2011; Perna et al., 2015; Batty et al., 2016; Lee et al., 2018). Several studies on cognitive impairment and mortality have found that elderly individuals with cognitive impairment have an increased risk of all-cause and cardiovascular mortality. Additionally, when cognitive impairment is accompanied by cardiovascular risk factors such as diabetes, chronic kidney disease, high blood pressure, or hypotension, the risk of mortality further increases (Zhu and Liao, 2020). Moreover, a study on cognitive impairment and mortality among elderly Chinese individuals suggested a dose–response relationship between baseline cognitive function and mortality. There was a monotonic increase in mortality risk observed across the range of cognitive severity (An and Liu, 2016). Our study found an association between cognitive impairment and an increased risk of long-term all-cause and cardiovascular mortality, consistent with previous research findings. However, our study did not analyze the relationship between cognitive impairment and cancer-related mortality. Previous research has indicated that tumor patients undergoing systemic therapy or radiotherapy may experience a decline in neurocognitive abilities (Olson and Marks, 2019). Under cognitive impairment, patients’ adherence to treatment may decrease compared to those without impairment, thereby increasing cancer-related mortality. Although the exact mechanisms linking peripheral tumors and cognitive impairment remain elusive, it can be speculated that there is a mutual interaction and influence between the two. Further research is needed to explore the related mechanisms.

Increasing evidence supports the role of mitochondrial dysfunction and increased oxidative stress in the pathogenesis of neurodegenerative diseases such as Alzheimer’s disease (AD). Both *in vitro* and *in vivo* experimental models have demonstrated the relevance of oxidative stress in dementia pathogenesis. The Mediterranean diet, known for its antioxidant properties including essential fatty acids, polyphenols in olive oil, and vitamins, may offer protective benefits (Cenini et al., 2019; Jurcau, 2021). The cumulative effects of the Mediterranean diet (MedD) on cardiometabolic health and glucose metabolism—both risk factors for cognitive decline—as well as the diet’s nutritional density, may influence cognitive outcomes through multiple mechanisms (Georgoulis et al., 2014; Stranges et al., 2019). An observational study covering 2016–2021 indicated that adherence to the MD positively impacts both cognitively impaired and unimpaired older adults, especially concerning their memory, in both the short and long term (Klimova et al., 2021). Additionally, changes in Alzheimer’s disease biomarkers, such as beta-amyloid (Aβ) deposition, tau phosphorylation, cortical thickness, or brain glucose metabolism, can occur 10–20 years before the clinical symptoms of AD appear (Vassilaki et al., 2018). A meta-analysis investigating the relationship between diet and hallmark AD biomarkers (tau and beta-amyloid) found that most studies on the MD indicate that adhering to it can significantly reduce the burden of AD biomarkers (Hill et al., 2019). Despite the current lack of robust clinical trial evidence, we still believe in the beneficial effects of the Mediterranean diet on cognitive function. Larger clinical trials are needed to further explore this potential.

Current research indicates that both the Mediterranean diet and cognitive impairment significantly impact mortality, particularly regarding all-cause mortality and cardiovascular death risk. Higher adherence to the Mediterranean diet has been shown to have a better effect on overall cognitive performance in older adults. This raises a question: do the Mediterranean diet and cognitive impairment jointly influence long-term mortality risk, and is there an interaction between the two?

Previous studies, such as those by Féart (2009) and Moustafa et al. (2022), have provided critical insights into the association between Mediterranean diet adherence and cognitive outcomes, including cognitive decline and dementia incidence. While these works established the protective role of the Mediterranean diet in neurodegeneration, our study extends this paradigm by focusing on mortality outcomes, a clinically significant yet underexplored endpoint in this context. Notably, our analysis revealed a novel interaction between low adherence to the Mediterranean diet and cognitive impairment in elevating cardiovascular mortality risk, a finding not previously reported in these prior studies. By integrating mortality outcomes, interaction effects, and a focus on vulnerable populations, our findings provide actionable evidence for tailoring dietary recommendations to mitigate excess mortality risk in older adults with cognitive impairment.

There may be a bidirectional relationship between adherence to the Mediterranean diet and cognitive abilities. Cognitive ability is closely linked to health literacy; individuals with lower cognitive abilities often find it challenging to maintain a healthy lifestyle. This can also affect their ability to adhere to the Mediterranean diet (Sabia et al., 2010; Kochan et al., 2017). Additionally, vascular dementia, a major component of cognitive impairment, has been shown in several studies to benefit from the Mediterranean diet’s positive effects on risk factors such as insulin resistance, inflammation, and endothelial dysfunction (Esposito et al., 2004; Shai et al., 2008). The interaction between the two can exacerbate disease severity and increase mortality risk for patients. Therefore, our study explored the interaction between adherence to the Mediterranean diet and cognitive impairment on long-term mortality outcomes. The results indicated that individuals with low adherence to the Mediterranean diet and cognitive impairment had higher risks of all-cause and cardiovascular mortality. Further subgroup analysis showed that only in individuals with cognitive impairment was higher adherence to the Mediterranean diet associated with reduced cardiovascular mortality risk. Low adherence to the Mediterranean diet and cognitive impairment exhibited a synergistic effect in increasing the risk of cardiovascular-related mortality. Our use of a continuous, validated aMED score and nationally representative NHANES data improves the robustness of observed associations compared to studies relying on binary dietary assessments or homogeneous cohorts. The dose–response relationship observed via restricted cubic splines (Figure 2) further supports biological plausibility, suggesting that even incremental improvements in MedDiet adherence may reduce mortality risk, particularly in cognitively vulnerable populations. Therefore, this study highlights the importance of the Mediterranean diet for individuals with cognitive impairments. Whether for prevention or treatment, the Mediterranean diet holds significant value in improving cognitive impairments.

However, this study has several limitations. First, since the research involved regression analysis with adjustment for various covariates, missing data for some covariates may affect the robustness of the results. Second, due to the cross-sectional design of NHANES, the causal relationship between the Mediterranean diet and cognitive impairment cannot be determined. Third, detailed information on smoking intensity (e.g., cigarettes per day) was unavailable in the NHANES dataset due to substantial missing data (>80%), precluding analysis of dose–response relationships. Fourth, the assessment of adherence to the Mediterranean diet was based on a 24-h dietary recall questionnaire, which could introduce recall bias and affect the accuracy of the results.

## 5 Conclusion

Adherence to the Mediterranean diet and cognitive impairment correlate in relation to cardiovascular-related mortality, but not all-cause mortality. In individuals with cognitive impairment, the effect of adherence to the Mediterranean diet on cardiovascular-related mortality is more pronounced. Future research and clinical practice could focus more on the dietary patterns of those with cognitive impairment to reduce disease severity.

## Data Availability

Publicly available datasets were analyzed in this study. This data can be found at: https://wwwn.cdc.gov/nchs/nhanes/default.aspx.

## Footnotes

^1^ https://www.cdc.gov/nchs/nhanes/irba98.htm

^2^ http://www.R-project.org

^3^ https://wwwn.cdc.gov/nchs/nhanes/tutorials/sampledesign.aspx

^4^ https://wwwn.cdc.gov/nchs/nhanes/analyticguidelines.aspx#sample-design
