# Supplementary material for: Correlation of cognitive impairment with Mediterranean diet and mortality: a prospective cohort study
Source: Front Aging Neurosci. 2025 Apr 9;17:1556608. doi: 10.3389/fnagi.2025.1556608 (PMC12014611; doi:10.3389/fnagi.2025.1556608)
Supplement: Supplementary file 1 [file Table_1.docx]

Supplementary Material

# Supplementary Tables

**Supplementary Table 1. Baseline demographics between patients with or without cognitive impairment.**

|  | **Without Cognitive impairment** | **With Cognitive impairment** | **P-value** |
| --- | --- | --- | --- |
| **Age (year)** | 68.1 (67.6 ,68.6) | 73.5 (72.6 ,74.3) | <0.0001 |
| **Female** | 55.1 (52.0 ,58.1) | 45.5 (38.9 ,52.2) | 0.0185 |
| **Race** |  |  | <0.0001 |
| **Mexican American** | 3.2 (2.0 ,4.9) | 4.4 (2.0 ,9.2) |  |
| **Other Hispanic** | 3.6 (2.4 ,5.3) | 5.6 (3.3 ,9.4) |  |
| **Non-Hispanic White** | 81.0 (77.1 ,84.3) | 71.1 (62.0 ,78.7) |  |
| **Non-Hispanic Black** | 7.2 (5.1 ,10.1) | 14.0 (9.9 ,19.5) |  |
| **Other Race** | 5.1 (4.0 ,6.6) | 4.9 (3.2 ,7.4) |  |
| **Family PIR** | 3.3 (3.1 ,3.5) | 2.7 (2.5 ,2.9) | 0.0001 |
| **Educational level** |  |  | 0.7622 |
| **Less than 9th grade** | 5.3 (3.9 ,7.3) | 6.1 (3.5 ,10.5) |  |
| **9-11th grade** | 9.7 (7.2 ,13.0) | 8.6 (5.9 ,12.5) |  |
| **High school graduate** | 21.6 (17.8 ,26.0) | 25.0 (20.2 ,30.6) |  |
| **College** | 30.5 (26.8 ,34.4) | 28.4 (23.0 ,34.4) |  |
| **College graduate or above** | 32.8 (27.6 ,38.5) | 31.9 (24.4 ,40.4) |  |
| **Marital status** |  |  | 0.0008 |
| **Married** | 68.5 (65.5 ,71.3) | 59.1 (51.5 ,66.3) |  |
| **Never married** | 3.8 (2.9 ,5.1) | 3.2 (1.9 ,5.1) |  |
| **Widowed/Divorced/Separated** | 27.7 (24.8 ,30.7) | 37.7 (31.1 ,44.9) |  |
| **Smoking** | 19.1 (16.8 ,21.5) | 18.9 (14.0 ,25.1) | 0.9578 |
| **BMI** | 29.2 (28.7 ,29.8) | 28.6 (27.8 ,29.4) | 0.2295 |
| **CHD** | 9.3 (7.2 ,11.8) | 12.6 (8.7 ,17.7) | 0.0845 |
| **Stroke** | 4.7 (3.6 ,6.1) | 11.4 (8.7 ,14.8) | <0.0001 |
| **Diabetes mellitus** | 23.1 (20.3 ,26.1) | 33.9 (29.5 ,38.6) | 0.0001 |
| **Hypertension** | 28.4 (24.8 ,32.2) | 16.6 (12.9 ,21.1) | <0.0001 |
| **TG (mmol/L)** | 1.4 (1.3 ,1.5) | 1.3 (1.2 ,1.4) | 0.2136 |
| **TC (mmol/L)** | 5.0 (5.0 ,5.1) | 4.7 (4.6 ,4.8) | <0.0001 |
| **HDL-C (mmol/L)** | 1.4 (1.4 ,1.5) | 1.4 (1.3 ,1.5) | 0.112 |
| **LDL-C (mmol/L)** | 2.9 (2.8 ,3.0) | 2.7 (2.5 ,2.8) | 0.0036 |
| **Fasting Glucose (mmol/L)** | 6.2 (5.9 ,6.4) | 6.3 (6.1 ,6.6) | 0.2661 |
| **HbA1c, %** | 5.9 (5.8 ,5.9) | 6.1 (6.0 ,6.2) | 0.0087 |
| **HOMA-IR** | 4.0 (3.4 ,4.7) | 3.8 (3.2 ,4.5) | 0.518 |
| **Albumin** | 42.1 (41.8 ,42.4) | 41.6 (41.2 ,42.0) | 0.043 |
| **eGFR, mL/min** | 73.4 (71.7 ,75.0) | 66.7 (64.4 ,69.1) | 0.0003 |

Data are presented as mean (95% CI) or percentage (95% CI). For continuous variables, p value was by survey-weighted linear regression (svyglm). For categorical variables, p value was by survey-weighted Chi-square test (svytable).

**Supplementary Table 2. VIF results of confounders included in the multivariate regression models.**

| Confounders | VIF results |
| --- | --- |
| Age | 1.1 |
| Gender | 1.1 |
| Race | 1.1 |
| Education | 1.3 |
| Marrital status | 1.2 |
| BMI | 1.2 |
| Smoking sttatus | 1.2 |
| FIR | 1.4 |
| Stroke | 1 |
| Diabetes | 1.1 |
| Hypertension | 1.1 |
